# Basic Emollients for Xerosis Cutis in Atopic Dermatitis: A Review of Clinical Studies

**DOI:** 10.1111/ijd.17793

**Published:** 2025-04-23

**Authors:** Andreas Wollenberg, Sebastien Barbarot, Antonio Torrelo

**Affiliations:** ^1^ Department of Dermatology and Allergy Augsburg University Hospital Augsburg Germany; ^2^ Comprehensive Center for Inflammation Medicine University of Luebeck Luebeck Germany; ^3^ Department of Dermatology and Allergy University Hospital, Ludwig‐Maximilian University Munich Germany; ^4^ Department of Dermatology Nantes Université, CHU Nantes, UMR 1280 PhAN, INRAE Nantes France; ^5^ Department of Dermatology Hospital Infantil Universitario Niño Jesús Madrid Spain

**Keywords:** atopic dermatitis, dry skin, eczema, emollient, moisturizer, xerosis cutis

## Abstract

Xerosis cutis (dry skin) is a common and burdensome symptom of atopic dermatitis (AD). Topical emollients restore skin hydration and barrier function through the physicochemical properties of their nonactive constituents (e.g., glycerol, urea, lactic acid, liquid paraffin, petrolatum) and represent the mainstay of basic therapy for xerosis cutis associated with AD. Newer “emollients plus” containing active ingredients may expand the treatment options available to patients with AD; however, we believe that basic emollients remain an important strategy for the long‐term management of xerosis cutis. To that end, this article aims to review the clinical value of basic emollients for treating xerosis cutis in AD. We performed a series of literature searches to identify clinical studies of basic emollients containing one or more of the following ingredients: almond and coconut oils, amino acids, chondroitin, dexpanthenol, glucose, glycerol, glycosaminoglycans, hyaluronic acid, lactic acid, lanolin, olive oil, paraffin, petrolatum, phospholipids, polyunsaturated fatty acids, pyroglutamic acid, squalene, triglycerides, urea, vegetable oils, and vitamin E. From these searches, the authors identified articles of interest that described the efficacy of basic emollients for the treatment of xerosis cutis associated with AD. Studies included in our review varied widely in terms of sample size, study design, interventions, and endpoints but collectively showed that most basic emollient formulations are safe and effective at improving objective and subjective measures of xerosis cutis. These studies also demonstrated the importance of ongoing emollient therapy to avoid xerosis relapse and the additive benefits of emollients that combine ingredients with complementary biophysical properties (e.g., glycerol with its humectant effect plus petrolatum with its occludent effect). Overall, the current body of literature reinforces the role of basic emollients as effective and accessible treatment options for the long‐term management of xerosis cutis in patients with AD.

**Summary**

- Why was the study undertaken?
  ○ We performed a series of literature searches to evaluate the clinical effectiveness of basic emollients for treating xerosis cutis (dry skin) associated with atopic dermatitis.
- What does this study add?
  ○ Our review summarizes a large body of evidence demonstrating the benefits of basic emollients on objective and subjective skin hydration and barrier function measures and the additive effects of combining ingredients with complementary biophysical properties.
- What are the implications of this study for disease understanding or clinical care?
  ○ Despite the development of more advanced therapies, current literature reinforces that basic emollients are safe, effective, and accessible treatment options for the long‐term management of xerosis cutis in patients with atopic dermatitis.

## 1 Introduction

Atopic dermatitis (AD) is the most common chronic inflammatory skin disease, with a prevalence of up to 20% among children and 2%–8% among adults worldwide [1]. The pathophysiology of AD is not fully understood; however, genetic predisposition, exogenous triggers, and endogenous factors are thought to contribute to defective skin barrier function and cutaneous inflammation characteristic of AD [2]. Xerosis cutis (dry skin) is common in patients with AD. European clinical guidelines recognize the importance of restoring skin barrier function in patients with AD and advocate that regular emollient use is the mainstay of treatment for xerosis cutis associated with this disease [3, 4].

Traditionally, emollients are topical vehicle‐type preparations that contain, as functionally relevant ingredients, a humectant or moisturizer to promote stratum corneum (SC) hydration (e.g., glycerol, urea, lactic acid) and an occludent to reduce evaporative water loss (e.g., liquid paraffin, petrolatum). Many emollient formulations contain water and other auxiliary substances (e.g., physiological lipids and antipruritic agents) [5, 6]. However, clinical guidelines from 2018 onward have differentiated between these “basic emollients” which exert biophysical effects through their nonactive constituents, and “emollients plus” which combine an emollient with nonmedicated, active ingredients (e.g., plant extracts, bacterial lysates) [1, 4, 7].

Although “emollients plus” formulations may provide new management options for patients with AD, we recognize that basic emollients still represent an important, effective, and lower‐cost strategy for the long‐term management of xerosis cutis. Indeed, experts agree that sufficient amounts of basic emollients are more beneficial in AD than insufficient amounts of more advanced, but also more costly, emollients plus [7]. Herein, we present a review of the current literature to evaluate the clinical benefits of basic emollients for xerosis cutis in patients with AD.

## 2 Methods

Literature searches were conducted to identify clinical studies describing the efficacy of basic emollients containing one or more of the following ingredients: almond and coconut oils, amino acids, chondroitin, dexpanthenol, glucose, glycerol, glycosaminoglycans, hyaluronic acid, lactic acid, lanolin, olive oil, paraffin, petrolatum, phospholipids, polyunsaturated fatty acids, pyroglutamic acid, squalene, triglycerides, urea, vegetable oils, and vitamin E. Key search terms included the ingredient of interest, in addition to “xerosis”, “dry skin”, “atopic dermatitis”, “ichthyosis”, “psoriasis”, “diabetic foot”, and relevant synonyms for each term (Table S1). Embase and Ovid MEDLINE database searches were performed in July 2022 and included articles published in 1974 (Embase searches) and 1946 (MEDLINE searches).

Emollient ingredients of interest were identified from a keyword search of relevant literature describing emollient therapy for xerosis cutis and diseases associated with dry skin. Active ingredients in “emollients plus” products were excluded from this review. Pure lipophilic oils and certain emollient lipophilic constituents that are naturally occurring in the skin (e.g., ceramides, cholesterol, fatty acids), when not combined with a humectant, do not fulfill the definition of a basic emollient, and studies describing these products were also excluded. Outcomes of interest included objective and subjective measures of xerosis cutis (e.g., skin capacitance, transepidermal water loss [TEWL], patient‐reported skin dryness); secondary outcomes including disease severity, related symptoms (e.g., pruritus) and tolerance were also reported. Studies that primarily examined the effect of emollients on other endpoints (e.g., preventing AD flares) were excluded from this review but are discussed by Vestergaard and colleagues later in this supplement [8].

Articles selected for inclusion in this review were based on the authors‘ expertise in the area, and additional articles of interest were retrieved through *ad hoc* literature searches. The present article discusses the utility of basic emollients for the management of xerosis cutis associated with AD; evidence describing basic emollients for other xeroses (particularly those associated with ichthyosis, psoriasis, and diabetic foot) is reviewed by Szepietowski and Tadini in the following article of this supplement [9].

## 3 Results and Discussion

In total, 21 literature searches were conducted (Table S1), yielding 28 articles for inclusion in this review (Table 1). No articles of interest were identified that described clinical studies of basic emollients containing amino acids, chondroitin, dexpanthenol, glucose, lanolin, olive oil, pyroglutamic acid, squalene, vegetable oils, or vitamin E for the management of xerosis cutis in patients with AD.

**TABLE 1:** Articles of interest describing clinical studies of basic emollients for xerosis cutis in patients with atopic dermatitis.

Ingredient(s).	Article of interest	Study type^a^	Population	Intervention	Key findings
Glycerol	Breternitz [10]	A: Placebo‐controlled, double‐blind, randomized, phase 3 study	24 patients with AD	AS: 20% glycerol AC: Aqua, cetearyl alcohol, isopropyl myristate, paraffinum liquidum, PEG‐40 hydrogenated castor oil, glyceryl behenate, glyceryl dibehenate, tribehenin, citric acid, sodium citrate, methylparaben, propylparaben CS: None (vehicle control) CC: Same as AC Applied twice daily for 4 weeks	Glycerol cream was associated with improvements in SC hydration (higher capacitance) and epidermal barrier function (lower TEWL) versus vehicle control. No differences in erythema values, skin pH, SCORAD, and local severity scores were detected between groups
	Lodén [11]	A/B: Randomized, double‐blind, parallel‐group study	109 patients with AD	AS: 20% glycerine AC: NR CS_1_: None (vehicle control) CC_1_: NR (same as AC) CS_2_: 4% urea, 4% sodium chloride CC_2_: NR Applied for 30 days	Urea cream was associated with lower TEWL versus glycerine cream and its vehicle control. Clinical assessment of dryness showed that urea cream was superior to glycerine. No difference in skin capacitance was found between groups
	Lodén [12]	A/B: Randomized double‐blind study	197 patients with AD	AS: 20% glycerine AC: Aqua, petrolatum, canola, mineral oil, cetearylalcohol, glycerylstearate, dime thicone, PEG‐100 stearate, glyceryl polymethacrylate, cholesterol, propyleneglycol, methylparaben, propylparaben CS_1_: Water (vehicle control) CC_1_: Same as AC CS_2_: 4% urea, 4% sodium chloride CC_2_: Paraffinum liquidum, PEG‐5‐glycerylstearate, cetylalcohol, stearyl alcohol, stearic acid, trometamol, methylparaben, propylparaben, hydrochloric acid, water Applied at least once daily for 30 days	Urea cream was associated with higher rates of patient‐reported smarting versus glycerine cream and its vehicle control. No differences in stinging, itching, irritation, improvements in skin dryness, and DASI scores between the glycerine and urea groups
Urea	Lodén [11]	Described above (see glycerol)			
	Lodén [12]	Described above (see glycerol)			

	Fredriksson [13]	B: Two randomized, double‐blind comparison studies	30 patients with AD and 30 patients with eczematous hand dermatitis	AS: 10% urea (Aquacare HP) AC: Multisterols, phospholipids, fatty diols (pH 6) CS: 10% urea (Calmurid) CC: Betaine, lactic acid (pH 3) Applied twice daily for 4 weeks	Investigators and patients both expressed a greater preference for the pH 6 urea cream based on efficacy and cosmetic acceptability. The sensation of burning upon application was reported in a higher number of patients treated with the pH 3 urea cream versus the pH 6 cream
	Pigatto [14]	B: Single‐blind study	30 healthy subjects and 40 patients with AD	AS: 10% urea (Laceran) AC: NR CS: None (Essex Base Cream) CC: White petrolatum, paraffin, cresol chloride, polyethyleneglycol Applied twice daily for 1 month	In patients with AD, urea cream was associated with improvements in dryness, itching, erythema, TEWL, and subjective feelings of skin plasticity versus base cream. Urea cream was also associated with reduced epidermal desquamation and changes in epidermal lipid composition (lower polar fatty acids, higher ceramides, and higher linoleic acid versus before treatment)
Bissonnette [15]	B: Randomized, double‐blind study	100 patients with AD	AS: 5% urea AC: Aqua/water, butyrospermum parkII/shea butter, glycerin, cyclohexasiloxane, paraffinum liquidum/mineral oil, sodium lactate, cetearyl alcohol, PEG‐100 stearate, glyceryl stearate, propylene glycol, glycine, tocopherol, stearic acid, myristic acid, palmitic acid, bisabolol, triethanolamine, dimethicone, dimethiconol, disodium EDTA, hydroxyethylpiperazine ethane sulfonic acid, xanthan gum, acrylates/C10‐30 alkyl acrylate crosspolymer, citric acid, chlorhexidine digluconate, phenoxyethanol, methylparaben, propylparaben, fragrance CS: 10% urea CC: Aqua, sodium lactate, paraffinum liquidum, octyldodecanol, caprylic/capric triglyceride, isopropyl palmitate, glycerin, PEG‐7 hydrogenated castor oil, benzyl alcohol, methoxy PEG‐22/docecyl glycol copolymer, PEG‐45/dodecyl glycol copolymer, dimethicone, magnesium sulfate, lactic acid, ozokerite, PEG‐2 hydrogenated castor oil, sorbitan isostearate, hydrogenated castor oil Applied twice daily for 42 days	Both urea creams were associated with improvements in SCORAD scores, with no significant differences between groups. Patient‐reported cosmetic acceptability was greater for the 5% versus 10% urea cream

	Freitag [16]	C: Open, multicenter, drug monitoring survey	1611 patients with AD, contact eczema, dry eczema, psoriasis, and pruritus	AS: 5% urea, 3% polidocanol (laurylmacrogol) AC: NR Applied for 4 weeks	Urea cream was associated with improvements in dry skin symptoms (dry, scaly, rough, itching) versus baseline. Overall skin status was judged by doctors and patients to be “good” or “very good” in almost 89% of cases at the end of treatment, versus 75% at baseline
	Lodén [17]	C: Single‐blind study	15 patients with AD	AS: 5% urea (Canoderm) AC: Aqua (water), caprylic/capric triglyceride, propylene glycol, hydrogenated canola oil, cetearyl alcohol, glyceryl polymethacrylate, dimethicone, paraffin, sodium lactate, carbomer, glyceryl stearate, PEG‐100 stearate, polysorbate 60, cetyl acetate, oleyl acetate, acetylated lanolin alcohol, lactic acid, propylparaben, methylparaben Applied twice daily for 20 days	Urea cream was associated with improvements in skin capacitance, TEWL, and susceptibility to SLS versus baseline
Andersson [18]; Lodén [19]	B: Double‐blind, randomized study	48 patients with AD	AS: 4% urea, 4% sodium chloride (Fenuril) AC: Liquid paraffin, PEG‐5‐glyceryl stearate, cetyl alcohol, stearyl alcohol, stearic acid, trometamol, methyl parahydroxybenzoate, propyl parahydroxybenzoate, hydrochloric acid, water CS: 5% urea (Canoderm) CC: Fractionated coconut oil, cetearyl alcohol, PEG‐20 stearate, hydrogenated canola oil, propylene glycol, carbomer, dimethicone, hard paraffin, glyceryl polymethacrylate, propylparaben, methylparaben, sodium lactate, lactic acid, glyceryl stearate, polyoxethylene stearate, cetyl acetate, oleyl acetate, acetylated lanolin alcohols, water Applied at least once daily for 30 days	Both urea creams were associated with improvements in dry skin severity (DASI score) and patient‐reported skin dryness/irritation, with no significant differences between groups. Significantly fewer patients experienced smarting sensations with the 5% versus 4% urea cream

Faergemann [20]	B: Randomized, single‐blind study	56 patients with AD	AS: 20% propylene glycol (Propyless) AC: NR CS: 4% urea, 4% sodium chloride (Fenuril) CC: NR Applied twice daily for 2 weeks	Propylene glycol cream was associated with less itching and irritation versus urea cream. Investigator‐assessed dry skin severity (DASI score) was not significantly different between groups
	Palombo [21]	B: Prospective, randomized, open, parallel‐group study	36 patients with AD	AS_1_: 5 mg/g chitosan‐based anti‐inflammatory compound (Atobiol) AC_1_: Tocotrienols, hyaluronic acid AS_2_: 5 mg/g chitosan‐based anti‐inflammatory compound (Atobiol) AC_2_: Aqua (water), hydrogenated polydecene, propylene glycol, pentylene glycol, cetyl PEG/PPG‐10/1 dimethicone, tocotrienols CS: Petrolatum CC: NR Applied twice daily for 1 week	All three creams were associated with improvements in erythema, scaling, crusting, pruritus, skin hydration, and TEWL versus baseline; active treatments were associated with greater improvements in some endpoints versus control
	Soma [22]	B: Left–right comparison study	28 patients with AD	AS: 2% nicotinamide AC: Behenyl alcohol, squalane, isocetyl myristate, octyldodecyl myristate, cholesterol, hydrogenated lecithin, glycerin, carbomer, water CS: White petrolatum CC: NR Applied twice daily for 8 weeks	Nicotinamide cream was associated with greater improvements in TEWL, SC hydration, and desquamation index versus white petrolatum
	Matsumoto [23]	B: NR	186 healthy subjects and 39 patients with AD	AS_1_: 3% heparinoid (Hirudoid) AC_1_: Glycerin, white petrolatum, lanolin, alcohol, thymol, methylparahydroxybenzoate, isopropanol AS_2_: 0.5% equine glycoceramides (AK‐cream) AC_2_: Glycerin, squaline, black sugar extract, citric acid, yeast extract, xanthan gum, butylene glycol CS: Pure petrolatum CC: NR Applied 4 times daily for 2 days	All three creams were associated with increased skin capacitance versus baseline

	Schario [24]	B: Participant‐blinded, randomized, prospective study	38 patients with dry skin and atopic predisposition	AS_1_: Ice plant ( *Mesembryanthemum crystallinum* ) extract (Ice Plant Body Care Lotion) AC_1_: Aqua, glycerin, alcohol, *simmondsia chinensis* oil, persea gratissima oil, prunus *amygdalus dulcis* oil, *manihot utilissima* starch, cera alba, lanolin, lysolecithin, *mangifera indica* seed butter, butyrospermum parkii butter, daucus carota extract, sucrose stearate, sucrose distearate, *chondrus crispus* extract, glyceryl stearate, hectorite, xanthan gum, stearic acid, *amyris balsamifera* oil, *Rosmarinus officinalis* extract, sodium stearoyl lactylate AS_2_: Ice plant ( *Mesembryanthemum crystallinum* ) extract (Intensive Ice Plant Cream) AC_2_: Aqua, persea gratissima oil, glycerin, *mangifera indica* seed butter, alcohol, tricaprylin, prunus *amygdalus dulcis* oil, *simmondsia chinensis* seed oil, *sesamum indicum* seed oil, lanolin, cetearyl alcohol, bentonite, butyrospermum parkii butter, daucus carota sativa root extract, rosmarinus officinalis leaf extract, *amyris balsamifera* bark oil, lysolecithin, glyceryl oleate, xanthan gum CS: Petrolatum CC: Aqua, propylene glycol, caprylic/capric triglyceride, glyceryl stearate, alcohol, cetyl alcohol, PEG‐20 glyceryl stearate Applied daily for 16 weeks	All three creams were associated with improved skin condition (SCORAD score) versus baseline, with no significant differences between groups. The ice plant extract creams demonstrated higher SC hydration values and significantly lower TEWL values versus petrolatum
Sawatzky [25]	B: Randomized study	65 patients with dry skin and atopic predisposition (modified Erlanger Atopy Score ≥ 4)	AS: Ice plant ( *Mesembryanthemum crystallinum* ) extract (Ice Plant Body Care Lotion and Intensive Ice Plant Cream) AC: NR CS: Petrolatum (DAC basic cream) CC: NR Applied daily for 16 weeks	Both treatments were associated with improvements in SCORAD index and skin scaliness (using microtopography) versus baseline, with no significant differences between groups
Glycerol and paraffin	August [26]	C: Prospective, single‐arm study	116 patients with eczema/AD (76%), psoriasis (6%), or other dry skin conditions (18%)	AS: 25% paraffin, 5% glycerine (Epaderm) AC: NR Applied at least twice daily for 4 weeks	Paraffin‐glycerine cream was associated with improvements in patient‐reported skin moisturization and softness, and clinical measures of xerosis and skin hydration versus baseline
	Boralevi [27]	A: Phase 3, multicenter, double‐blind, randomized, vehicle‐controlled study	251 patients with AD	AS: 15% glycerol, 10% liquid and soft paraffin (Dexeryl) AC: Glycerol monostearate, stearic acid, polydimethylcyclosiloxane, silicone oil, macrogol 600, trolamine, propyl parahydroxybenzoate, purified water CS: None CC: Glycerol monostearate, stearic acid, polydimethylcyclosiloxane, silicone oil, macrogol 600, trolamine, propyl parahydroxybenzoate, purified water Applied twice daily for 28 days (double‐blind period) and up to 84 days (open‐label period)	During the double‐blind period, the glycerol‐paraffin/petrolatum cream was associated with greater improvements in AD severity, xerosis, pruritus, and skin hydration versus control. During the open‐label period, AD relapse occurred after stopping emollient treatment, but improvement returned when treatment resumed
	Tiplica [28]	B: Randomized, open‐label study	335 patients with AD	AS: 15% glycerol, 10% liquid, and soft paraffin (Dexeryl) AC: NR CS_1_: NR (Atopiclair) CC_1_: NR CS_2_: None (no emollient) CC_2_: None Applied 2–3 times daily for 12 weeks	Both creams were associated with greater improvements in AD severity (SCORAD, PO‐SCORAD, POEM) versus no emollient
	Cristaudo [29]	C: Open‐label, prospective study	50 patients with AD	AS: Glycerin, paraffin (Dexeryl) AC: NR Applied twice daily for 2 months	Glycerin‐paraffin cream was associated with improvements in skin xerosis, fissuring, itching, erythema, TEWL, skin hydration, and DLQI scores versus baseline
Lactic acid and almond oil	Simon [30]	B: Randomized, double‐blind, study	50 patients with xerotic eczema including AD	AS: 5% lactic acid, 10% refined almond oil, 20%–30% linoleic acid (Antidry Lotion) AC: NR CS: 5% lactic acid, 10% refined almond oil, 5% polidocanol (Antidry Calm) CC: NR Applied twice daily for 14 days	Both creams were associated with improvements in itching severity, skin moisture, and lipid content versus baseline
Hyaluronic acid	Lee [31]	C: Prospective, single‐arm study	25 patients with AD	AS: 0.01% H.ECM liposomes (soluble proteoglycan, hydrolyzed collagen, hyaluronic acid) AC: NR Applied twice daily for 4 weeks	The liposome cream was associated with improvements in itching severity, TEWL, and skin hydration versus baseline
Glycosaminoglycans	Lee [31]	Described above (see hyaluronic acid)			
	Kawakami [32]	C: Questionnaire	110 patients with AD	AS: Heparinoid mucopolysaccharide (95% of patients) AC: NR Applied twice daily for 1 month	All patients responded that emollients were at least somewhat effective for treating their dry skin, and most reported that emollients were at least somewhat effective at treating their pruritus or eczematous skin
Phospholipids	Tamura [33]	B: Randomized, controlled study	Patients with AD	AS: Phospholipid AC: Glycerin, butylene glycol, squalane, cholesterol, and cetyl ethylhexanoate CS: 3.0 mg/g heparinoid (Hirudoid) CC: Glycerin, squalane, petrolatum, cetyl alcohol, carbomer Applied twice daily for 4 weeks	Both creams were associated with improvements in skin findings, dryness and desquamation score, pruritus score, TEWL, and moisture content, with no significant differences between groups
Polyunsaturated fatty acids	Eberlein [34]	C: Multinational, multicentre, observational, noncontrolled, prospective cohort study	2456 patients with AD	AS: 0.3% palmitoylethanolamine (Physiogel A.I.) AC: Purified water, Olea Europeaea, glycerol, pentylene glycol, palm glycerides, Olus, hydrogenated lecithin, Squalane, betaine, sarcosine, acetamide MEA, hydroxyethylcellulose, sodium carbomer, carbomer, Xanthan Gum Applied at least twice daily for 4–6 weeks	The palmitoylethanolamine cream was associated with improvements in skin dryness, erythema, pruritus, excoriation, scaling, lichenification, sleep quality, and topical corticosteroid use versus baseline
	Yang [35]	B: Three randomized, parallel‐group controlled trials	181 patients with AD, chronic eczema, or pruritus hiemalis	AS: Ceramides, linoleic acid (YuZe Skin Barrier Recovery Body Lotion) plus 0.1% mometasone furoate (Elocon) AC (emollient): Safflower seed oil, rice bran oil CS: 0.1% mometasone furoate (Elocon) CC: NR Applied for 2 months	In patients with AD, the combination of ceramide‐linoleic acid cream and topical glucocorticoid was associated with greater improvements in skin capacitance, TEWL, pruritus intensity, and eczema severity versus topical glucocorticoid alone
Triglycerides	Danby [36]	B: Double‐blind, intrasubject, vehicle‐controlled, single open‐application study	22 patients with AD	AS_1_: Ceramides, triglycerides, cholesterol (CeraVe cream) AC_1_: Aqua/water, glycerin, cetearyl alcohol, caprylic/capric triglyceride, cetyl alcohol, ceteareth‐20, petrolatum, dimethicone, phenoxyethanol, behentrimonium methosulfate, potassium phosphate, ethylhexylglycerin, sodium lauroyl lactylate, disodium EDTA, dipotassium phosphate, ceramide NP, ceramide AP, phytosphingosine, cholesterol, xanthan gum, carbomer, sodium hyaluronate, tocopherol, ceramide EOP AS_2_: Ceramides, triglycerides, cholesterol (CeraVe lotion) AC_2_: Aqua/water, glycerin, caprylic/capric triglyceride, cetearyl alcohol, cetyl alcohol, dimethicone, phenoxyethanol, polysorbate 20, ceteareth‐20, behentrimonium methosulfate, polyglyceryl‐3 diisostearate, sodium lauroyl lactylate, ethylhexylglycerin, potassium phosphate, disodium EDTA, dipotassium phosphate, ceramide NP, ceramide AP, phytosphingosine, cholesterol, xanthan gum, carbomer, sodium hyaluronate, tocopherol, ceramide EOP CS_1_: Liquid paraffin, white soft paraffin (Zerobase) CC_1_: Purified water, cetostearyl alcohol, cetomacrogol, sodium dihydrogen phosphate, chlorocresol, phosphoric acid CS_2_: White soft paraffin, liquid paraffin (Epimax) CC_2_: Purified water, polysorbate 60, cetosteryl alcohol, phenoxyethanol CS_3_: White soft paraffin (Aquamax) CC_3_: Purified water, cetostearyl alcohol, liquid paraffin, polysorbate 60, phenoxyethanol CS_4_: None (no emollient) CC_4_: None	The test cream and lotion were each associated with increased skin hydration over 24 h versus the reference creams and no treatment control. All test and reference creams were associated with reductions in visual skin dryness over 24 h versus the no treatment control
	Danby [37]	B: Randomized observer‐blind intrapatient‐controlled study	34 patients with AD	AS: Ceramides, triglycerides, cholesterol (CeraVe) AC: Aqua/water, glycerin, cetearyl alcohol, caprylic/capric triglyceride, cetyl alcohol, ceteareth‐20, petrolatum, dimethicone, phenoxyethanol, behentrimonium methosulfate, potassium phosphate, ethylhexylglycerin, sodium lauroyl lactylate, disodium EDTA, dipotassium phosphate, ceramide NP, ceramide AP, phytosphingosine, cholesterol, xanthan gum, carbomer, sodium hyaluronate, tocopherol, ceramide EOP CS: 11% liquid paraffin, 10% white soft paraffin (Zerobase) CC: Chlorocresol, cetomacrogol, cetostearyl alcohol, phosphoric acid, sodium dihydrogen phosphate, purified water Applied twice daily for 28 days	The test cream was associated with greater improvements in TEWL, SC lipid structure, skin barrier integrity, skin hydration, and visual signs of dryness versus the reference cream

Abbreviations: AC, active carrier; AD, atopic dermatitis; AS, active substance; CC, control/comparator carrier; CS, control/comparator substance; DASI, Dry skin Area and Severity Index; DLQI, Dermatology Life Quality Index; EDTA, ethylenediaminetetraacetic; NR, not reported; POEM, Patient Oriented Eczema Measure; PO‐SCORAD, Patient‐Oriented SCORing Atopic Dermatitis index; SC, stratum corneum; SCORAD, SCORing Atopic Dermatitis index; SLS, sodium laurel sulfsate; TEWL, transepidermal water loss.

^a^ Study types were labeled as either A (emollient containing an ingredient of interest was compared with its vehicle control), B (emollient containing an ingredient of interest was compared with a different emollient formulation or comparator), or C (other; neither A nor B applies).

### 3.1 Emollient Constituents With Hydrating Properties

#### 3.1.1 Glycerol

Glycerol is a trihydroxy alcohol and a common constituent of topical emollients due to its hygroscopic properties that improve SC hydration and skin barrier function [38]. These effects were demonstrated in a double‐blind, randomized, phase 3 study by Breternitz and colleagues, in which 24 patients with AD applied a 20% glycerol cream or glycerol‐free placebo to their left or right forearms twice daily for 4 weeks [10]. At 4 weeks, treatment with the glycerol‐based emollient was associated with significant improvements in SC hydration (as measured by skin capacitance) and numerical improvements in epidermal barrier function (as measured by TEWL) when compared with placebo [10].

A randomized, double‐blind study by Lodén and colleagues found that changes in TEWL and skin capacitance were not significantly different between 40 patients who applied a 20% glycerol cream for 30 days versus 34 patients randomized to an otherwise identical, glycerol‐free control [11]. Compared with the glycerol‐treated group, TEWL was lower, and clinical assessment of skin dryness was improved in a third group of 35 patients who received a cream containing 4% urea and 4% sodium chloride [11]. However, a similarly designed study of 197 patients found that smarting was less common among those treated with the glycerol‐ versus urea‐based cream and that improvements in skin dryness (as assessed by both patients and dermatologists) were similar between the two emollients [12]. Other studies of emollients for AD that contain glycerol in combination with paraffin are described below.

### 3.2 Urea

Urea is a common constituent of topical dermatology products, including emollients for AD (Table 1), and it exerts hydrating, keratolytic, and antipruritic effects depending on the concentration applied [39]. An early randomized, double‐blind study by Fredriksson and Gip compared the efficacy of two urea‐based emollients in 60 patients with AD or eczematous hand dermatitis [13]. Patients received one of two 10% urea creams; one with a pH of approximately 6 (Aquacare HP, AbbVie, United States) and the other with a pH of approximately 3 (Calmurid, Galderma, Switzerland) [13]. After 4 weeks of twice‐daily treatment to left or right body areas, more investigators and patients expressed a preference for the pH 6 urea cream over the pH 3 cream on the basis of efficacy, and the pH 6 urea cream was associated with comparatively less burning [13]. Similarly, Pigatto and colleagues demonstrated that twice‐daily application of a 10% urea cream (Laceran, Beiersdorf AG, Germany) was associated with subjective improvements in xerosis cutis, pruritus, and erythema among patients with AD, greater reductions in TEWL versus a petrolatum/paraffin‐based reference cream (Essex Base Cream, Schering‐Plow, United States), and reduced epidermal desquamation leading to improved skin barrier function [14].

More recently, a randomized, double‐blind study by Bissonnette and colleagues compared a 5% urea moisturizer with a 10% urea lotion in 100 patients with AD but showed that clinical improvements with emollient treatment were not significantly different between the test and reference creams [15]. However, this study also found that most patients preferred using the 5% urea moisturizer over the 10% urea lotion based on their responses to a cosmetic acceptability questionnaire [15]. An extensive, postmarketing drug monitoring survey evaluated the safety and efficacy of a cream containing 5% urea and 3% polidocanol in 1611 patients with AD, contact eczema, dry eczema, psoriasis, and pruritus [16]. Over 4 weeks of treatment, the urea‐based cream was found to be well tolerated, alleviated symptoms including scaling, dryness, roughness, and itching, and improved overall skin status as assessed by patients and physicians [16].

Several articles of interest have described clinical studies of a 5% urea cream (Canoderm, ACO Hud AB, Sweden) for managing AD [17, 18, 19]. Lodén and colleagues found that twice‐daily application of 5% urea cream for 20 days increased skin capacitance, reduced TEWL, and improved skin susceptibility to irritants among 15 patients with AD [17]. In a double‐blind, randomized study of 48 patients that compared the 5% urea cream with one containing 4% urea and 4% sodium chloride (Fenuril, Pharmacia & Upjohn AB, Sweden), both creams were associated with patient‐ and dermatologist‐reported improvements in xerosis severity, but fewer patients reported skin sensations (i.e., smarting, stinging, itching) with the 5% urea cream [18, 19]. Skin irritation with the 4% urea‐sodium chloride cream was similarly reported by Faergemann and colleagues, who showed that a lotion containing 20% propylene glycol (Propyless, Schering‐Plow, Belgium) may be less irritating but as effective as the urea cream in patients with AD [20].

Overall, these studies provide insight into the large body of clinical evidence to support the effectiveness of urea‐based emollients for the management of xerosis cutis associated with AD. The data suggest that formulations with a lower urea concentration (i.e., 5% vs. 10%) or a more neutral pH (i.e., pH 6 vs. pH 3) may be more cosmetically acceptable among patients with AD but without loss of efficacy.

### 3.3 Emollient Constituents With Film‐Forming Properties

#### 3.3.1 Petrolatum

Petrolatum is a semi‐solid mixture of mostly long‐chain hydrocarbons and a common occludent in topical emollient therapies. Most petrolatum‐related articles of interest in this review have described studies that used petrolatum products as controls. For example, a prospective, randomized, parallel‐group trial by Palombo and colleagues evaluated the efficacy of a chitosan‐derived compound (Atobiol, manufacturer unknown) in a polysaccharide‐based lamellar emulsion or a carrier emulsion versus petrolatum ointment in 36 patients with AD [21]. Similarly, Soma and colleagues describe a right–left comparison study that evaluated the moisturizing effects of a 2% nicotinamide cream versus white petrolatum in 28 patients with AD [22]. In another study of 37 infants with AD, changes in skin capacitance were compared between patients treated with 3% heparinoid cream (Hirudoid, Maruho, Japan), 0.5% equine glycoceramide cream (AK‐cream, Rosette, Japan) or pure petrolatum [23]. Data from these studies indicated some benefit of the test products over the petrolatum‐based controls but nevertheless reinforced the positive effects of petrolatum‐containing emollients on outcomes, including TEWL, SC hydration, and clinical appearance of dryness and scaling [21, 22, 23].

Two articles of interest described a randomized trial that compared emollients containing ice plant ( *Mesembryanthemum crystallinum* ) extract with those containing petrolatum in children at risk of developing AD [24, 25]. After daily use for 16 weeks, some SC hydration and TEWL values were significantly improved with the ice plant‐based emollient versus petrolatum; however, SCORing of Atopic Dermatitis (SCORAD) index scores and skin dryness were significantly improved in all patients, regardless of emollient used [24]. In a related analysis, daily use of the ice plant‐ and petrolatum‐based emollients was associated with significant reductions in subjective and objective SCORAD scores among patients and quality of life improvements as reported by their parents [25].

Taken together, these studies suggest that classical petrolatum‐based emollients remain an effective treatment option for AD; however, their greasy, occlusive properties may limit their cosmetic acceptability and long‐term use by patients [40].

### 3.4 Combinations of Key Emollient Constituents

#### 3.4.1 Glycerol and Paraffin

Most emollients are complex formulations that typically combine a water‐binding humectant (e.g., glycerol, urea, lactic acid) with a film‐forming occludent (e.g., liquid paraffin, petrolatum) to leverage the biophysical properties of individual constituents [4]. For example, all articles of interest for emollients containing paraffin were clinical studies that evaluated products co‐formulated with glycerol (Table 1).

Recently, the efficacy and safety of an emollient cream containing 25% paraffin and 5% glycerol (Epaderm, Mölnlycke Health Care AB, Sweden) were assessed in a single‐arm study of 114 participants, 76% of whom had eczema or AD [26]. Patient‐reported skin moisturization, softness, and pruritus were significantly improved after 4 weeks of at least twice‐daily use, and clinical measures of xerosis cutis and skin hydration were also improved considerably [26].

**FIGURE 1:**
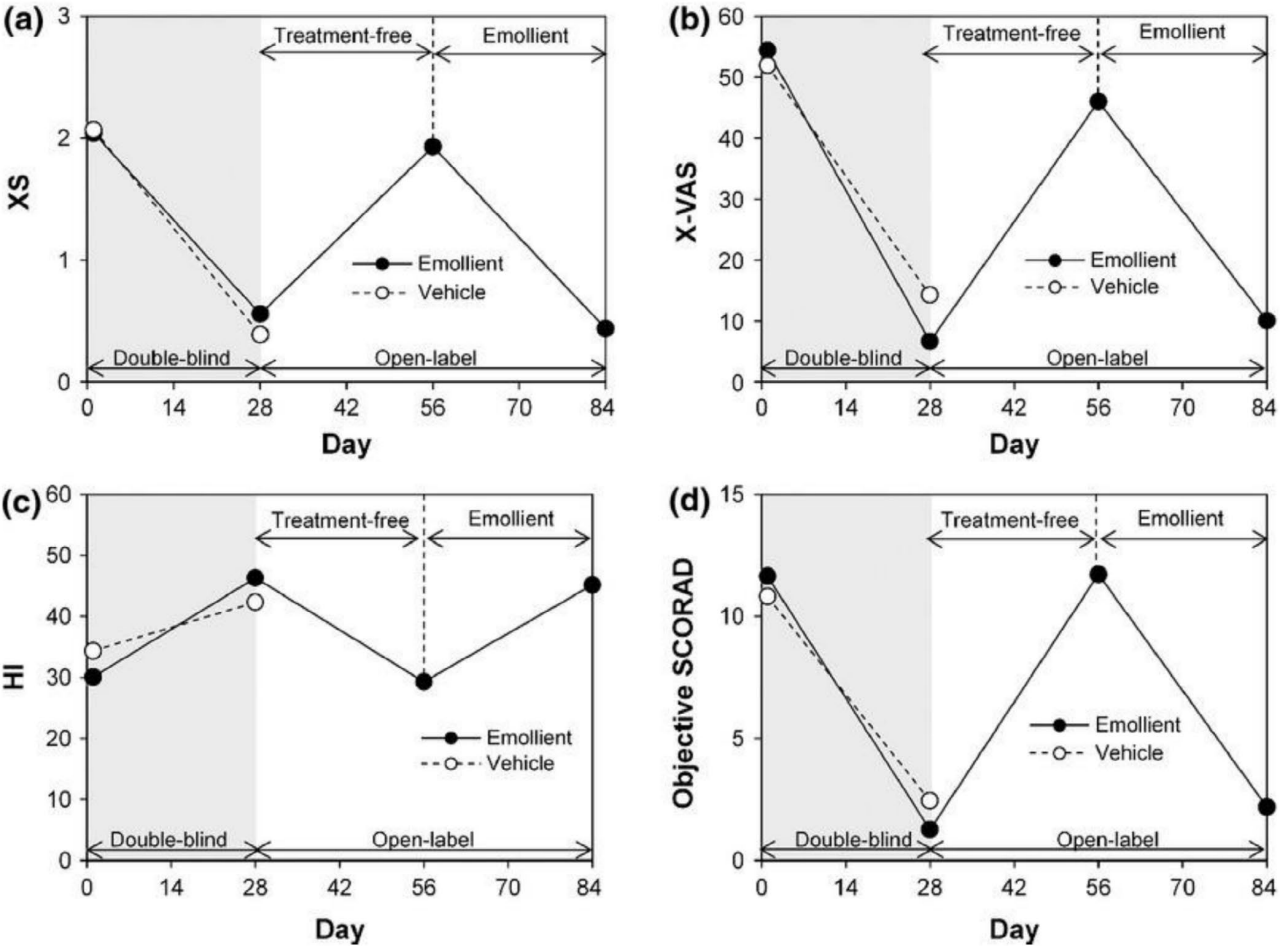
Changes in mean SCORAD xerosis score (a), mean xerosis visual analog scale score (b), mean skin hydration index (c), and mean objective SCORAD score (d) during a phase 3 study of a 15% glycerol and 10% liquid paraffin/petrolatum emollient in children with atopic dermatitis [27]. In this study, patients (*n* = 251) were randomized to apply study emollient or vehicle control twice daily during a 28‐day double‐blind period. Nonresponders on day 28 received study emollient until day 84 (open‐label period); responders on day 28 stopped emollient treatment until day 56 when they were reassessed and resumed study treatment until day 84 if relapse had occurred. Data are shown for responders who worsened and resumed study treatment on day 56. HI, hydration index; SCORAD, SCORing of Atopic Dermatitis index; XS, xerosis score; X‐VAS, xerosis visual analog scale score. Figure reproduced with permission from Boralevi F, et al. *J Eur Acad Dermatol Venereol* 2014; 28: 1456–62. 2013 John Wiley and Sons. All rights reserved.

The efficacy and tolerability of an emollient containing 15% glycerol and 10% liquid paraffin/petrolatum (Dexeryl, Pierre Fabre Medicament, France) were assessed in an international, phase 3, multicenter, double‐blind, randomized, vehicle‐controlled trial of 251 children with AD and moderate xerosis cutis [27]. In this study, the glycerol‐liquid paraffin/petrolatum cream (or its vehicle control) was applied twice daily during a 28‐day double‐blind period, then open‐label treatment with active emollient was continued up to day 84 depending on patient response [27]. During the double‐blind period, the glycerol‐liquid paraffin/petrolatum cream was associated with greater improvements in overall disease severity (measured by SCORAD index scores), xerosis cutis (measured using the SCORAD xerosis score and visual analog score) and skin hydration (measured by corneometry) (all *p* < 0.001 vs. vehicle control; Figure 1) [27]. Moreover, the open‐label period demonstrated the long‐term tolerability of the glycerol‐liquid paraffin/petrolatum cream and showed that: (1) in the absence of emollient treatment, xerosis severity returned rapidly to baseline levels (Figure 1) and (2) clinical improvements were achievable in patients who did not initially respond to emollient treatment [27]. These data highlight that emollients should be applied regularly and continuously in patients with AD.

A subsequent, randomized, open‐label study of the glycerol‐liquid paraffin/petrolatum cream in 335 children with AD similarly found that emollient treatment for 12 weeks improved AD severity as assessed by physicians and parents [28]. An open‐label study by Cristaudo and colleagues evaluated the effects of the glycerol‐liquid paraffin/petrolatum cream on instrumental, clinical, and subjective measures of treatment response in 50 patients with AD [29]. After 60 days of twice‐daily application, emollient therapy was associated with significant improvements in biophysical parameters (TEWL and skin hydration), which coincided with significant improvements in disease severity (measured by SCORAD index and four‐point scales for xerosis, fissuring, itching, and erythema) and patient quality of life [29]. Overall, these studies consistently demonstrate that emollients combining glycerol and paraffin/petrolatum represent an effective strategy for managing xerosis cutis in AD patients.

#### 3.4.2 Lactic Acid and Almond Oil

The effectiveness of topical lactic acid, a component of a natural moisturizing factor in the skin, was evaluated in a randomized, double‐blind study of two lactic acid‐containing emollients in 50 patients with xerotic eczema, including AD [30]. In this intraindividual study, emollients containing 5% lactic acid, 10% refined almond oil, and 20%–30% linoleic acid (Antidry Lotion, Merz Pharma (Schweiz) AG, Switzerland) and 5% lactic acid, 10% refined almond oil, and 5% polidocanol (Antidry Calm, Merz Pharma (Schweiz) AG, Switzerland) were applied to left and right body sides twice daily for 14 days [30]. Both emollients were well tolerated and were associated with significant improvements in patient‐reporting itching severity, skin moisture, and lipid content [30].

### 3.5 Other Constituents of Basic Emollients

#### 3.5.1 Hyaluronic Acid and Glycosaminoglycans

Glycosaminoglycans, including hyaluronic acid, are long, hygroscopic polysaccharide chains and a major structural component of the skin's extracellular matrix [41]. A prospective, single‐arm study by Lee and colleagues sought to assess the safety and efficacy of a novel liposome emollient (H.ECM, Hugel, Korea) containing a soluble proteoglycan with hydrolyzed collagen and hyaluronic acid [31]. In this study, 25 patients with AD applied the emollient twice daily for 4 weeks and demonstrated significant improvements in skin barrier function (as measured by changes in TEWL and skin hydration from baseline) and patient‐reported itching severity over the treatment period [31].

A questionnaire conducted by Kawakami and Soma aimed to elicit patient views on the effectiveness of emollients for AD management in Japan [32]. The questionnaire was completed by 103 patients with AD, 95% of whom used heparinoid mucopolysaccharide creams or lotions to manage their condition [32]. After 1 month of twice‐daily use, all patients responded that emollients were at least somewhat effective at treating their dry skin, and most reported that emollients were at least somewhat effective at treating their pruritus or eczematous skin [32].

#### 3.5.2 Phospholipids, Polyunsaturated Fatty Acids, and Triglycerides

In addition to humectants and occludents, topical emollients may contain lipids that replenish the intercellular lipid bilayer of the SC and increase the skin's barrier function [5, 6]. The effectiveness of a phospholipid‐containing moisturizing cosmetic in patients with AD was assessed in a randomized clinical trial by Tamura and colleagues [33]. Improvements in skin findings, dryness and desquamation score, pruritus score, TEWL, and SC hydration were observed following 4 weeks of twice‐daily use, and improvements with the phospholipid‐containing cosmetic were comparable to those observed with a heparinoid‐based control product (Hirudoid lotion, Maruho, Japan) [33].

The international, prospective, observational ATOPA study evaluated the effect of an emollient containing 0.3% palmitoylethanolamine (Physiogel A.I. Cream, Stiefel Laboratories, Germany) in 2456 patients with AD [34]. After at least twice‐daily application for 4–6 weeks, treatment was associated with significant improvements in objective and subjective outcomes, including skin dryness, erythema, pruritus, excoriation, scaling, lichenification, sleep quality, and topical corticosteroid use [34].

Yang and colleagues demonstrated the benefit of a ceramide‐ and linoleic acid‐containing moisturizer (YuZe Skin Barrier Recovery Body Lotion, Shanghai Jahwa United Company, China) as an adjunctive agent for the management of AD, chronic eczema, and pruritus hiemalis [35]. In this study, patients were assigned to treatment with the ceramide‐linoleic acid moisturizer plus 0.1% mometasone furoate cream or mometasone furoate cream alone for 2 months [35]. The moisturizer and topical corticosteroid application were found to rapidly re‐establish skin barrier function and ameliorate pruritus in patients with AD and pruritus hiemalis but did not produce the same effect in those with chronic eczema [35].

Two articles of interest evaluated the barrier‐restoring effects of a multivesicular emulsion containing ceramides, triglycerides, and cholesterol (CeraVe, L'Oreal, France) in patients with dry, AD‐prone skin [36, 37]. In a double‐blind study of 22 patients, a single application of the test cream and lotion was each associated with significant improvements in SC hydration and skin dryness compared with no treatment and achieved sustained moisturization over 24 h compared with three paraffin‐based reference creams (Zerobase, Thornton & Ross, United Kingdom; Epimax, Dermato‐Logical, United Kingdom; and Aquamax, Intrapharm Laboratories, United Kingdom) [36]. In a subsequent observer‐blind, randomized, intrapatient‐controlled study of 34 patients, twice‐daily application of the test cream for 28 days was associated with reduced TEWL, improved SC lipid structure and skin barrier integrity, increased skin hydration, and decreased visual signs of dryness compared with the Zerobase reference cream [37].

## 4 Conclusions

The articles of interest identified in this review highlight the clinical value of basic emollients for managing xerosis cutis in patients with AD. Our review summarizes a large body of evidence that consistently demonstrates the benefits of common emollient constituents on objective and subjective measures of skin hydration and barrier function, as well as the additive effects of combining ingredients with complimentary biophysical properties (e.g., glycerol with its humectant effect plus petrolatum with its occludent effect). Most studies in our review evaluated the effectiveness of emollients applied at least once daily for 4 weeks or longer, with one study showing that xerosis severity can return to baseline levels after cessation of emollient treatment (Figure 1) [27]. Together, these data highlight the importance of frequent and ongoing emollient therapy for the long‐term management of xerosis cutis in patients with AD.

The findings of this review complement those of Vestergaard and colleagues, who reviewed the wider spectrum of clinical benefits associated with emollient therapy for dry skin and xerotic conditions, including AD [8]. Together, our observations are consistent with previous systematic reviews of emollient therapy for AD and eczema, which found that most formulations were associated with clinical benefits, including disease severity improvements, prevention of flares, and reductions in corticosteroid use [42, 43]. However, these reviews and the present study collectively highlight a paucity of large, high‐quality, head‐to‐head, randomized trials to evaluate the comparative efficacy of one emollient over another [42, 43]. Nevertheless, the current body of literature reinforces that basic emollients are safe, effective, and accessible treatment options for the long‐term management of xerosis cutis in patients with AD.

## Conflicts of Interest

A.W. has served as an advisor, speaker or investigator for AbbVie, Aileens Pharma, Almirall, Amgen, Beiersdorf, Bioderma, Bristol Myers Squibb, Eli Lilly, Galapagos, Galderma, Glenmark, GSK, Hans Karrer, Janssen, LEO Pharma, L'Oreal, Maruho, Merck (MSD), Novartis, Pfizer, Pierre Fabre, Regeneron, Sanofi‐Aventis, Sandoz, and UCB. S.B. reports support for the present manuscript from Pierre Fabre; payment or honoraria for lectures, presentations, speakers' bureaus, manuscript writing or educational events from AbbVie, Alexion, Almirall, AstraZeneca, Eli Lilly, Galderma, Incyte, Janssen, LEO Pharma, Novartis, Pfizer, Sanofi‐Genzyme and UCB; support for meeting attendance or travel from AbbVie, Alexion, Almirall, Eli Lilly, Galderma, Janssen, LEO Pharma, Pfizer, Sanofi‐Genzyme and UCB; and has served on data safety monitoring or advisory boards for AbbVie and Sanofi‐Genzyme. A.T. has served as an advisor, speaker, or investigator for AbbVie, Eli Lilly, LEO Pharma, Novartis, Pfizer, Pierre Fabre, Sanofi, and Viatris.

## Supporting information


Data S1.
