# Supplementary material for: Basic Emollients for Xerosis Cutis in Atopic Dermatitis: A Review of Clinical Studies
Source: Int J Dermatol. 2025 Apr 23;64(Suppl 1):13–28. doi: 10.1111/ijd.17793 (PMC12124105; doi:10.1111/ijd.17793)
Supplement: Supplementary file 1 — Data S1. [file IJD-64-13-s001.docx]

## Supplementary Table S1.

Literature search strategies and articles of interest describing clinical studies of basic emollient ingredients for the management of xerosis cutis in patients with atopic dermatitis.

| **Ingredient(s)** | **Literature search strategy [number of articles]^a^** |
| --- | --- |
| **Almond and coconut oils** | Date of search: July 11, 2022   1. ((almond* adj4 oil*) or (coconut* adj4 oil*) or (coconut* adj4 butter*) or (coconut* adj4 fat*)).ti,ab,kw [6639] 2. exp almond oil/dt or exp coconut oil/dt [299] 3. (xerosis or xeroderma* or dry skin).ti,ab,kw [19,049] 4. exp xerosis/ or exp dry skin/ [14,918] 5. (atopic dermatitis or neurodermatitis or sulzberger or eczema or ichthyos?s or psoriasis or diabetic foot).ti,ab,kw [242,926] 6. exp atopic dermatitis/ or exp ichthyosis/ or exp psoriasis/ or exp diabetic foot/ [254,529] 7. (1 or 2) and (3 or 4) and (5 or 6) [20] 8. Remove duplicates from step 7 [15]   1 article of interest identified by the authors |
| **Amino acids** | Date of search: July 12, 2022   1. (amino acid* or aminoacid*).ti,ab,kw [1,081,207] 2. (xerosis or xeroderma* or dry skin).ti,ab,kw [19,056] 3. exp xerosis/ or exp dry skin/ [14,922] 4. (atopic dermatitis or neurodermatitis or sulzberger or eczema or ichthyos?s or psoriasis or diabetic foot).ti,ab,kw [243,098] 5. exp atopic dermatitis/ or exp ichthyosis/ or exp psoriasis/ or exp diabetic foot/ [254,695] 6. 1 and (2 or 3) and (4 or 5) [66] 7. Remove duplicates from step 6 [42]   No articles of interest identified by the authors |
| **Chondroitin** | Date of search: July 11, 2022   1. chondroitin*.ti,ab,kw [35,963] 2. exp chondroitin sulfate/dt [1526] 3. (xerosis or xeroderma* or dry skin).ti,ab,kw [19,049] 4. exp xerosis/ or exp dry skin/ [14,918] 5. (atopic dermatitis or neurodermatitis or sulzberger or eczema or ichthyos?s or psoriasis or diabetic foot).ti,ab,kw [242,926] 6. exp atopic dermatitis/ or exp ichthyosis/ or exp psoriasis/ or exp diabetic foot/ [254,529] 7. (1 or 2) and (3 or 4) and (5 or 6) [2] 8. Remove duplicates from step 7 [2]   No articles of interest identified by the authors |
| **Dexpanthenol** | Date of search: July 6, 2022   1. (dexpanthenol* or panthenol* or pantothenol* or pantothenyl* or $pantoylpropanolamin*).ti,ab,kw [1065] 2. exp dexpanthenol/dt [435] 3. (xerosis or xeroderma* or dry skin).ti,ab,kw [19,066] 4. exp xerosis/ or exp dry skin/ [14,924] 5. (atopic dermatitis or neurodermatitis or sulzberger or eczema or ichthyos?s or psoriasis or diabetic foot).ti,ab,kw [243,014] 6. exp atopic dermatitis/ or exp ichthyosis/ or exp psoriasis/ or exp diabetic foot/ [254,625] 7. (1 or 2) and (3 or 4) and (5 or 6) [11] 8. Remove duplicates from step 7 [10]   No articles of interest identified by the authors |
| **Glucose** | Date of search: July 8, 2022   1. (glucose or dextros*).ti,ab,kw [1,232,786] 2. exp glucose/dt [5823] 3. (xerosis or xeroderma* or dry skin).ti,ab,kw [19071] 4. exp xerosis/ or exp dry skin/ [14,950] 5. (atopic dermatitis or neurodermatitis or sulzberger or eczema or ichthyos?s or psoriasis or diabetic foot).ti,ab,kw [243,183] 6. exp atopic dermatitis/ or exp ichthyosis/ or exp psoriasis/ or exp diabetic foot/ [254,808] 7. (1 or 2) and (3 or 4) and (5 or 6) [24]   No articles of interest identified by the authors |
| **Glycerol** | Date of search: July 7, 2022   1. (glycerol or glycerin?).ti,ab,kw [114,858] 2. exp glycerol/dt [1915] 3. (xerosis or xeroderma* or dry skin).ti,ab,kw [19,068] 4. exp xerosis/ or exp dry skin/ [14,925] 5. (atopic dermatitis or neurodermatitis or sulzberger or eczema or ichthyos?s or psoriasis or diabetic foot).ti,ab,kw [243,098] 6. exp atopic dermatitis/ or exp ichthyosis/ or exp psoriasis/ or exp diabetic foot/ [254,698] 7. (1 or 2) and (3 or 4) and (5 or 6) [75] 8. Remove duplicates from step 7 [57]   7 articles of interest identified by the authors |
| **Glycosaminoglycans** | Date of search: July 11, 2022   1. (glycosaminoglycan* or GAG? or HSGAG? or CSGAG? or mucopolysaccharide* or polymucopolysaccharide*).ti,ab,kw [106,113] 2. exp glycosaminoglycan/dt [63,285] 3. (xerosis or xeroderma* or dry skin).ti,ab,kw [19,049] 4. exp xerosis/ or exp dry skin/ [14,918] 5. (atopic dermatitis or neurodermatitis or sulzberger or eczema or ichthyos?s or psoriasis or diabetic foot).ti,ab,kw [242,926] 6. exp atopic dermatitis/ or exp ichthyosis/ or exp psoriasis/ or exp diabetic foot/ [254,529] 7. (1 or 2) and (3 or 4) and (5 or 6) [28] 8. Remove duplicates from step 7 [26]   2 articles of interest identified by the authors |
| **Hyaluronic acid** | Date of search: July 6, 2022   1. (hyaluronic acid or hyaluronate* or hyaluronan).ti,ab,kw [78,324] 2. exp hyaluronic acid/dt [6182] 3. (xerosis or xeroderma* or dry skin).ti,ab,kw [19,066] 4. exp xerosis/ or exp dry skin/[14,924] 5. (atopic dermatitis or neurodermatitis or sulzberger or eczema or ichthyos?s or psoriasis or diabetic foot).ti,ab,kw [243,014] 6. exp atopic dermatitis/ or exp ichthyosis/ or exp psoriasis/ or exp diabetic foot/ [254,625] 7. (1 or 2) and (3 or 4) and (5 or 6) [23] 8. Remove duplicates from step 7 [20]   1 article of interest identified by the authors |
| **Lactic acid** | Date of search: July 7, 2022   1. ((lactic* adj1 acid*) or lactate or (hydrox* adj1 fatty acid*)).ti,ab,kw [350,793] 2. exp lactic acid/dt or exp hydroxy fatty acid/dt [1110] 3. (xerosis or xeroderma* or dry skin).ti,ab,kw [19,056] 4. exp xerosis/ or exp dry skin/ [14,922] 5. (atopic dermatitis or neurodermatitis or sulzberger or eczema or ichthyos?s or psoriasis or diabetic foot).ti,ab,kw [243,098] 6. exp atopic dermatitis/ or exp ichthyosis/ or exp psoriasis/ or exp diabetic foot/ [254,695] 7. (1 or 2) and (3 or 4) and (5 or 6) [67] 8. Remove duplicates from step 7 [54]   1 article of interest identified by the authors |
| **Lanolin** | Date of search: July 8, 2022   1. (lanolin* or wool yolk or wool wax or wool greas* or wool fat or woolfat).ti,ab,kw [1383] 2. exp lanolin/dt [164] 3. (xerosis or xeroderma* or dry skin).ti,ab,kw [19,071] 4. exp xerosis/ or exp dry skin/ [14,950] 5. (atopic dermatitis or neurodermatitis or sulzberger or eczema or ichthyos?s or psoriasis or diabetic foot).ti,ab,kw [243,183] 6. exp atopic dermatitis/ or exp ichthyosis/ or exp psoriasis/ or exp diabetic foot/ [254,808] 7. (1 or 2) and (3 or 4) and (5 or 6) [10]   No articles of interest identified by the authors |
| **Olive oil** | Date of search: July 7, 2022   1. ((olive adj3 oil*) or (olea adj4 oil*) or oleum olivarum or (glycer?l adj1 oleat*) or olivat*).ti,ab,kw [28,316] 2. exp olive oil/ [20,727] 3. (xerosis or xeroderma* or dry skin).ti,ab,kw [19,049] 4. exp xerosis/ or exp dry skin/ [14,918] 5. (atopic dermatitis or neurodermatitis or sulzberger or eczema or ichthyos?s or psoriasis or diabetic foot).ti,ab,kw [242,926] 6. exp atopic dermatitis/ or exp ichthyosis/ or exp psoriasis/ or exp diabetic foot/ [254,529] 7. (1 or 2) and (3 or 4) and (5 or 6) [23] 8. Remove duplicates from step 7 [18]   No articles of interest identified by the authors |
| **Paraffin** | Date of search: July 12, 2022   1. (paraffin* or (vaselin* adj3 oil*)).ti,ab,kw [144,357] 2. exp paraffin/dt [192] 3. (xerosis or xeroderma* or dry skin).ti,ab,kw [19,056] 4. exp xerosis/ or exp dry skin/ [14,922] 5. (atopic dermatitis or neurodermatitis or sulzberger or eczema or ichthyos?s or psoriasis or diabetic foot).ti,ab,kw [243,098] 6. exp atopic dermatitis/ or exp ichthyosis/ or exp psoriasis/ or exp diabetic foot/ [254,695] 7. (1 or 2) and (3 or 4) and (5 or 6) [36] 8. Remove duplicates from step 7 [26]   4 articles of interest identified by the authors |
| **Petrolatum** | Date of search: July 7, 2022   1. (petrolatum* or vaselin* or (soft* adj3 paraffin*)). ti,ab,kw [6313] 2. exp petrolatum /dt [816] 3. (xerosis or xeroderma* or dry skin).ti,ab,kw [19,065] 4. exp xerosis/ or exp dry skin/ [14,923] 5. (atopic dermatitis or neurodermatitis or sulzberger or eczema or ichthyos?s or psoriasis or diabetic foot).ti,ab,kw [242,878] 6. exp atopic dermatitis/ or exp ichthyosis/ or exp psoriasis/ or exp diabetic foot/ [254,548] 7. (1 or 2) and (3 or 4) and (5 or 6) [89] 8. Remove duplicates from step 7 [69]   5 articles of interest identified by the authors |
| **Phospholipids** | Date of search: July 12, 2022   1. (phospholipid* or phosphatide*).ti,ab,kw [227,279] 2. exp phospholipid/dt [2870] 3. (xerosis or xeroderma* or dry skin).ti,ab,kw [19,056] 4. exp xerosis/ or exp dry skin/ [14922] 5. (atopic dermatitis or neurodermatitis or sulzberger or eczema or ichthyos?s or psoriasis or diabetic foot).ti,ab,kw [243,098] 6. exp atopic dermatitis/ or exp ichthyosis/ or exp psoriasis/ or exp diabetic foot/ [254,695] 7. (1 or 2) and (3 or 4) and (5 or 6) [19] 8. Remove duplicates from step 7 [14]   1 article of interest identified by the authors |
| **Polyunsaturated fatty acids** | Date of search: July 7, 2022   1. (fatty acid* or PUFA? or omega-* or polyunsaturated fat*).ti,ab,kw [540,032] 2. exp polyunsaturated fatty acid/dt or exp fatty acid/dt [47,391] 3. (xerosis or xeroderma* or dry skin).ti,ab,kw [19,054] 4. exp xerosis/ or exp dry skin/ [14,921] 5. (atopic dermatitis or neurodermatitis or sulzberger or eczema or ichthyos?s or psoriasis or diabetic foot).ti,ab,kw [243,002] 6. exp atopic dermatitis/ or exp ichthyosis/ or exp psoriasis/ or exp diabetic foot/ [254,602] 7. (1 or 2) and (3 or 4) and (5 or 6) [125] 8. Remove duplicates from step 7 [105]   2 articles of interest identified by the authors |
| **Pyroglutamic acid** | Date of search: July 11, 2022   1. (pyroglutamic acid or pyroglutamate or PCA or 5-oxoproline or pidolic acid or pidolate).ti,ab,kw [133,924] 2. exp pyroglutamic acid/dt [65] 3. (xerosis or xeroderma* or dry skin).ti,ab,kw [19,049] 4. exp xerosis/ or exp dry skin/ [14,918] 5. (atopic dermatitis or neurodermatitis or sulzberger or eczema or ichthyos?s or psoriasis or diabetic foot).ti,ab,kw [242,926] 6. exp atopic dermatitis/ or exp ichthyosis/ or exp psoriasis/ or exp diabetic foot/ [254,529] 7. (1 or 2) and (3 or 4) and (5 or 6) [13] 8. Remove duplicates from step 7 [11]   No articles of interest identified by the authors |
| **Squalene** | Date of search: July 12, 2022   1. (squalane or perhydrosqualene).ti,ab,kw [711] 2. (xerosis or xeroderma* or dry skin).ti,ab,kw [19,056] 3. exp xerosis/ or exp dry skin/ [14,922] 4. (atopic dermatitis or neurodermatitis or sulzberger or eczema or ichthyos?s or psoriasis or diabetic foot).ti,ab,kw [243,098] 5. exp atopic dermatitis/ or exp ichthyosis/ or exp psoriasis/ or exp diabetic foot/ [254,695] 6. 1 and (2 or 3) and (4 or 5) [2] 7. Remove duplicates from step 6 [2]   No articles of interest identified by the authors |
| **Triglycerides** | Date of search: July 12, 2022   1. (triglyceride* or triacylglycerol or triacylglyceride*).ti,ab,kw [331,602] 2. exp triacylglycerol/dt [912] 3. (xerosis or xeroderma* or dry skin).ti,ab,kw [19,056] 4. exp xerosis/ or exp dry skin/ [14,922] 5. (atopic dermatitis or neurodermatitis or sulzberger or eczema or ichthyos?s or psoriasis or diabetic foot).ti,ab,kw [243,098] 6. exp atopic dermatitis/ or exp ichthyosis/ or exp psoriasis/ or exp diabetic foot/ [254,695] 7. (1 or 2) and (3 or 4) and (5 or 6) [49] 8. Remove duplicates from step 7 [37]   2 articles of interest identified by the authors |
| **Urea** | Date of search: July 7, 2022   1. (urea or carbamid* or carbonyldiamid* or diaminomethan*).ti,ab,kw [208,416] 2. exp urea/dt [1787] 3. (xerosis or xeroderma* or dry skin).ti,ab,kw [19,068] 4. exp xerosis/ or exp dry skin/ [14,925] 5. (atopic dermatitis or neurodermatitis or sulzberger or eczema or ichthyos?s or psoriasis or diabetic foot).ti,ab,kw [243,098] 6. exp atopic dermatitis/ or exp ichthyosis/ or exp psoriasis/ or exp diabetic foot/ [254,698] 7. (1 or 2) and (3 or 4) and (5 or 6) [186] 8. Remove duplicates from step 7 [149]   10 articles of interest identified by the authors |
| **Vegetable oils** | Date of search: July 11, 2022   1. ((vegetable* adj4 oil*) or (vegetable* adj4 fat*) or (vegetable* adj4 butter*) or (plant* adj4 oil*) or (seed* adj4 oil) or (sunflower* adj4 oil*) or (helianthus adj4 oil*) or oleum helianti?).ti,ab,kw [51,711] 2. exp vegetable oil/dt or exp sunflower oil/dt [9617] 3. (xerosis or xeroderma* or dry skin).ti,ab,kw [19,049] 4. exp xerosis/ or exp dry skin/ [14,918] 5. (atopic dermatitis or neurodermatitis or sulzberger or eczema or ichthyos?s or psoriasis or diabetic foot).ti,ab,kw [242,926] 6. exp atopic dermatitis/ or exp ichthyosis/ or exp psoriasis/ or exp diabetic foot/ [254,529] 7. (1 or 2) and (3 or 4) and (5 or 6) [61] 8. Remove duplicates from step 7 [53]   No articles of interest identified by the authors |
| **Vitamin E** | Date of search: July 11, 2022   1. (alpha-tocopher* or vitamin-E or tocopher*).ti,ab,kw [100,356] 2. exp alpha tocopherol/dt [16,200] 3. (xerosis or xeroderma* or dry skin).ti,ab,kw [19,049] 4. exp xerosis/ or exp dry skin/ [14,918] 5. (atopic dermatitis or neurodermatitis or sulzberger or eczema or ichthyos?s or psoriasis or diabetic foot).ti,ab,kw [242,926] 6. exp atopic dermatitis/ or exp ichthyosis/ or exp psoriasis/ or exp diabetic foot/ [254,529] 7. (1 or 2) and (3 or 4) and (5 or 6) [24] 8. Remove duplicates from step 7 [22]   No articles of interest identified by the authors |

^a^Literature searches were performed using Embase and Ovid MEDLINE databases and included articles published from 1974 (Embase searches) and from 1946 (MEDLINE searches).
